# Near-InfraRed Spectroscopy Provides a Reproducible Estimate of Muscle Aerobic Capacity, but Not Whole-Body Aerobic Power

**DOI:** 10.3390/s24072277

**Published:** 2024-04-03

**Authors:** Tomas Venckunas, Andrius Satas, Marius Brazaitis, Nerijus Eimantas, Saule Sipaviciene, Sigitas Kamandulis

**Affiliations:** 1 Institute of Sport Science and Innovations, Lithuanian Sports University, 44221 Kaunas, Lithuania; 2 Department of Health Promotion and Rehabilitation, Lithuanian Sports University, 44221 Kaunas, Lithuania

**Keywords:** aerobic power, mitochondria, NIRS, skeletal muscle, oxidative capacity, aerobic fitness, oxygen consumption, oxidative metabolism

## Abstract

Near-infrared spectroscopy (NIRS) during repeated limb occlusions is a noninvasive tool for assessing muscle oxidative capacity. However, the method’s reliability and validity remain under investigation. This study aimed to determine the reliability of the NIRS-derived mitochondrial power of the musculus vastus lateralis and its correlation with whole-body (cycling) aerobic power ($\dot{V}$O_2_ peak). Eleven healthy active men (28 ± 10 y) twice (2 days apart) underwent repeated arterial occlusions to induce changes in muscle oxygen delivery after 15 s of electrical muscle stimulation. The muscle oxygen consumption (m$\dot{V}$O_2_) recovery time and rate (*k*) constants were calculated from the NIRS O_2_Hb signal. We assessed the reliability (coefficient of variation and intraclass coefficient of correlation [ICC]) and equivalency (*t*-test) between visits. The results showed high reproducibility for the m$\dot{V}$O_2_ recovery time constant (ICC = 0.859) and moderate reproducibility for the *k* value (ICC = 0.674), with no significant differences between visits (*p* > 0.05). NIRS-derived *k* did not correlate with the $\dot{V}$O_2_ peak relative to body mass (*r* = 0.441, *p* = 0.17) or the absolute $\dot{V}$O_2_ peak (*r* = 0.366, *p* = 0.26). In conclusion, NIRS provides a reproducible estimate of muscle mitochondrial power, which, however, was not correlated with whole-body aerobic capacity in the current study, suggesting that even if somewhat overlapping, not the same set of factors underpin these distinct indices of aerobic capacity at the different (peripheral and whole-body systemic) levels.

## 1. Introduction

Whole-body maximal oxygen uptake ($\dot{V}$O_2_ max) is one of the most commonly used parameters of aerobic power and is directly associated with exercise capacity and inversely associated with mortality [1,2,3,4]. The $\dot{V}$O_2_ max depends on both the “central” (i.e., largely cardiac blood pumping capacity and oxygen carrying capacity of the blood) and the “peripheral” (i.e., muscle capacity to use oxygen) sets of factors [5,6]. As a major site for oxidative phosphorylation, skeletal muscle is an important player in metabolic health, and its mitochondrial oxidative capacity is negatively associated with the risk of cardiovascular disease [7].

Because of its high importance for overall health and working capacity, skeletal muscle mitochondrial power warrants appropriate investigation. While tissue analysis in vitro after surgical sampling (biopsy) has a role in determining the oxidative metabolic capacity of the muscle, its applicability is limited because of the inconvenience related to the invasiveness of the procedure. Noninvasive techniques such as magnetic resonance spectroscopy have enhanced the study of mitochondrial function, but this method is costly and requires a high level of technical expertise. Therefore, alternative, noninvasive methods to assess muscle mitochondrial capacity need to be developed. Near-infrared spectroscopy (NIRS) provides a noninvasive measure of muscle oxygenation [8,9]; therefore, calculating the kinetics of (de)oxygenized soft tissue hemoglobin during repeated high-precision blood flow occlusions together with metabolic activation of the muscle with contractile activity allows us to obtain an objective measure of skeletal muscle aerobic/oxidative capacity corresponding closely with values obtained by phosphorus magnetic resonance spectroscopy [8,9].

NIRS variables (i.e., recovery of oxyhemoglobin [HbO_2_], deoxyhemoglobin [HHb], or calculated tissue saturation index) have been investigated for different limb muscles including plantar flexors [10,11,12,13,14,15,16,17,18,19], plantar extensors [16], knee extensors [10,20], knee flexors [20], wrist flexors [11,17], and some other superficial small muscles [13] in different age and gender populations ranging from completely inactive [21] to highly trained individuals [22,23]. Many of these studies addressed the day-to-day reproducibility of the markers of mitochondria capacity and generated varying but overall promising results, especially those highlighted in more recent publications [20,24,25].

However, even with this increasingly widespread application, the methodology is far from robustly established yet, and further improvements are sought. For instance, before muscular oxygen kinetics are measured using NIRS and mitochondrial power is estimated, metabolic activation of the muscle is achieved with voluntary muscle contraction [9,26] or electrical stimulation [20], and recently, even the possibility of not activating the muscle by any contractions preceding the measurements has been proposed [27]. Some studies have been designed to intentionally cover a wide range of aerobic fitness of the participants, e.g., [13,15,17], making it questionable the reliability and validity of the methodology in more relevant settings of less heterogeneous groups as those meeting the physical activity guidelines. All the above and more detailed nuances of the methodology, such as the differences between and within the studies in the extent of the induced mitochondrial respiration, an important factor for the correctness of the measurements [28], add to the complexity of the findings and their interpretation and may lead to reported “somewhat large” test–retest variability [25].

Therefore, the issue of the reproducibility of NIRS-derived signals, their ability to represent true/real skeletal muscle mitochondrial capacity, and the physiological and practical significance of the indirectly derived measures for physiological whole-body functioning merit further evaluation [14]. In the present study, we tested the reliability of noninvasively (in situ) assessed mitochondrial respiration power over a short (2-day) period and its correlation with whole-body aerobic power measured via expired gas analysis (indirect calorimetry) during the exhaustive exercise of large muscle groups at the same site.

## 2. Materials and Methods

### 2.1. Participants

Recreationally active young adult men (*n* = 11; 28.1 ± 9.7 y; 181.5 ± 6.0 cm; 79.8 ± 14.1 kg; BMI 24.1 ± 3.0; skin color type: 1 to 2 on a 5-point scale) were recruited for this study. The number of participants was chosen based on the quantity of participants recruited in similar studies (*n* = 10–17) [10,20,26]. This facilitates direct comparisons between studies. Participants reported engaging in unstructured moderate-intensity aerobic exercise (such as jogging, swimming, basketball, or football playing) 1–3 times per week, with a duration of less than 120 min at moderate intensity and/or no more than 30 min at vigorous intensity per week. The exclusion criteria for the present study included musculoskeletal pain within the previous 6 months, cardiovascular, pulmonary, or metabolic diseases, or regular use of medication or dietary supplements. Participants were in self-reported good health, which was confirmed by a medical history, visual inspection, and physical examination. During the study, participants were directed to maintain their regular dietary and physical activity routines, with the instruction to sleep for at least 7 h the night before each test, abstain from alcohol, and exercise 48 h before trial day.

This study complied with the ethical principles for experiments with humans as described in the most recent revision of the Declaration of Helsinki. The Regional Biomedical Research Ethics Committee approved the study protocol (license no. BE-2-47). Written informed consent was obtained from all participants after an explanation of all the details of the experimental procedures and the associated discomforts and risks.

### 2.2. Study Organization

During the initial meeting in the lab, scheduled a week before the study, participants were introduced to the study’s objectives and experimental design. They received instructions regarding daily activity and dietary regimens. Participants were then familiarized with NIRS procedures, electrical muscle stimulation, and stationary cycling on an electrically braked ergometer through several submaximal cycling bouts.

This study consisted of two phases involving the same participants. In the first phase, participants visited the laboratory on two separate occasions, with a 2-day interval, for measurements of musculus vastus lateralis mitochondrial respiration using NIRS during repetitive occlusions following an electric muscle stimulation protocol. In the second phase, within one week, a cycling ramp test until exhaustion was conducted to measure whole-body aerobic power [29].

Measurements were consistently conducted at the same time of day to minimize the influence of circadian rhythms. Participants were allowed to drink still water as needed until 60 min before the experiment to standardize their hydration status. This study was conducted in a laboratory maintained at a temperature of 22 °C and a relative humidity of 40–60%. All procedures were carried out by the same team of investigators at the Institute of Sport Science and Innovations at Lithuanian Sports University. This study was partly blinded, as testing and analysis of the data were performed by different investigators.

### 2.3. Anthropometric and Body Composition Measurements

Body height was measured shoeless using a stadiometer (Martin, GPM instrument, Siber Hegner, Zurich, Switzerland) with a precision of 0.1 cm. The measures were taken to check the correct position of the head in a standard position. Body mass and fat measurements were conducted using an electronic body composition analyzer (Tanita TBF-300 UK Ltd., West Drayton, UK), featuring four electrodes beneath the feet. The device extrapolates overall body composition from impedance, calculated body fat, and fat-free mass using a proprietary equation based on resistance, weight, height, age, and sex [30].

### 2.4. Measurement of Mitochondrial Respiratory Capacity (NIRS Procedure)

Testing was performed according to the protocol described by Brizendine et al. [31]. Participants wore shorts and laid on a padded couch with their legs extended comfortably during the procedure. The participants rested motionless on the couch for at least 10 min. NIRS optodes (NIRSport2 NIRx Medical Technologies, Berlin, Germany; Acquisition Software Aurora fNIRS) were fixed with a strap on the alcohol-cleansed skin on the thigh of the dominant leg over the musculus vastus lateralis approximately two-thirds from its proximal origin and were covered with a blackout blanket to protect from ambient light. The NIRS had two channels (two light sources and two photodiode detectors), and laser diodes at 760 nm and 850 nm were used. The probes (transmitter and receiving optode) were set at a 40 mm distance, rendering a penetration depth of approximately 20 mm. Stimulation electrodes (45 × 98 mm fully gelled non-woven electrostimulation electrodes, FIAB, Italy) were adhered to the skin in close proximity (distally and proximally) to the optodes. A blood pressure cuff (SC12D; D.E. Hokanson, Bellevue, WA, USA) was placed proximally on the thigh close to the inguinal crease of the groin, and a rapid pressure regulation system (E20 Cuff Inflator; D.E. Hokanson) powered by a 10-gallon (38 L) commercially available air compressor was used for the fast arterial blood flow occlusions and (hyperemic) reperfusions.

**Figure 1:**
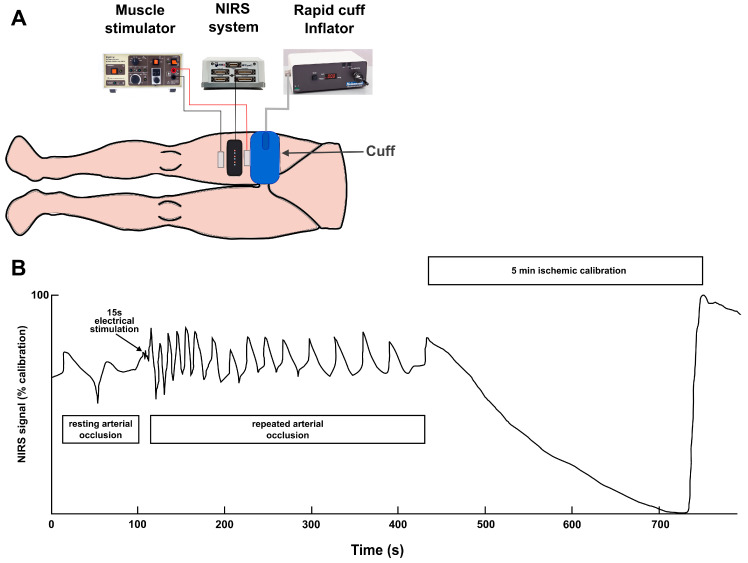
(**A**) NIRS procedure setup; (**B**) representative NIRS signal of the m. vastus lateralis obtained with the leg occlusion–reperfusion protocol used.

The test protocol (Figure 1) began with resting measurements by pressurizing the cuff to 250 mmHg to induce arterial blood flow occlusion. After 30 s, with blood flow still occluded, 15 s of stimulation with 200 μs biphasic 75 mV pulses (DS7AH; Digitimer, Welwyn Garden City, UK) followed, and then for the estimation of the muscular oxygen consumption rate (m$\dot{V}$O_2_) dynamics, a series of shorter cuff occlusions were immediately performed in the following order: 5 × 5 s (5 s relief), 5 × 10 s (10 s relief), and 5 × 15 s (15 s relief). Finally, the ischemic/hyperemia step for calibration purposes was conducted as follows: blood flow was occluded for 5 min to completely deoxygenate the muscle tissue under the optodes, and then the pressure was released to obtain the peak hyperemic response with maximal oxygenation [32].

Oxyhemoglobin (O_2_Hb) content was recorded at 35 Hz. The data were analyzed with MATLAB software (v. 9.4; MathWorks, Natick, MA, USA). m$\dot{V}$O_2_ was calculated as the initial 5 s downward slope of the change in the O_2_Hb signal during the arterial occlusion using a simple linear regression for each of the 15 occlusions. A monoexponential function was applied to derive the time and rate constants from the fifteen m$\dot{V}$O_2_ values. m$\dot{V}$O_2_ was calculated in absolute units using a path length factor of four, as suggested by the manufacturer.

m$\dot{V}$O_2_ was also expressed differentially as a percentage of the ischemic calibration per unit of time. The post-stimulation repeated measurements of m$\dot{V}$O_2_ were fitted to a monoexponential curve according to the following equation:

Y(x) = End − Δ × e^(−x*k)^

where Y is the relative m$\dot{V}$O_2_ during the arterial occlusion, End is the m$\dot{V}$O_2_ immediately after ceasing exercise, Δ is the change in m$\dot{V}$O_2_ from rest to end exercise, and k is the rate constant.

The correction of NIRS signals for changes in blood volume was performed as described by Ryan et al. [10]. The equation below describes the calculation of the correction factor:

β(t) = (|O_2_ Hb(t)|)/((|O_2_ Hb(t)| + |HHb(t)|)),

where β is the blood volume correction factor, t represents time, O_2_Hb is the oxygenated hemoglobin/myoglobin signal, and HHb is the deoxygenated hemoglobin/myoglobin signal. β is a nondirectional factor representing the proportionality of the change in blood volume (values range from 0 to 1).

The coefficient β was calculated for each data point. Once β was defined, its application to the raw data is shown below:

O_2_Hbc = O_2_Hb − [tHb × (1 − β)],

where O_2_Hbc is the corrected oxygenated hemoglobin/myoglobin signal, tHb is the arithmetic sum of the uncorrected O_2_Hb and HHb (the blood volume signal from the NIRS device), and β is the blood volume correction factor.

### 2.5. Whole-Body Aerobic Capacity ($\dot{V}$O_2_ Peak) Test

Before the testing procedure, the participants completed a warm-up composed of 5 min of dynamic stretching exercises for the lower body and a 5 min submaximal intensity pedaling cycle ergometer (Excalibur Sport; Lode, Groningen, The Netherlands) at about 70 rpm, with the external resistance corresponding to the power, whose number of watts corresponded to the participant’s body weight in kg to the nearest 5 W.

The cycling ramp test on an electrically braked ergometer (Excalibur Sport; Lode, Groningen, The Netherlands) was started at 40 W, and the resistance for pedaling increased by ramping to give a 30 W/min (1 W/2 s) increase in power. Volume and composition of the expired air were measured using a stationary spirometer operating in breath-by-breath mode (MetaLyzer 3B, Cortex Biophysik, Leipzig, Germany), with the gas analyzer’s CO_2_ and O_2_ sensors calibrated properly prior to each testing session with room air and factory gas mixtures of known composition. Heart rate (HR) was continuously monitored via a chest strap heart rate monitor (Polar H10, Polar Electro Oy, Kempele, Finland) and averaged at 5 s intervals.

Participants pedaled at 70 rpm until they were exhausted, which was considered an inability to sustain a pedaling cadence above 60 rpm for 10 consecutive seconds despite strong verbal encouragement. Additional two criteria for test validity were a respiratory exchange ratio (RER, which is VCO_2_/$\dot{V}$O_2_) of >1.10 attained at the end of the test protocol and the rate of perceived exertion of at least 19 points on the modified Borg scale. Absolute whole-body aerobic power was considered the highest oxygen consumption rate ($\dot{V}$O_2_ peak) during the test, calculated over the 20 s period using a rolling average (L/min). The body mass-scaled $\dot{V}$O_2_ peak (mL/kg/min) was also used for subsequent analyses as an index of relative whole-body aerobic power and is presented in Table 1. The highest averaged per 5 s HR (HR peak), highest pulmonary ventilation (VE), and highest RER, both averaged per 20 s, along with peak power output (PPO) achieved during the test, are also presented in Table 1.

### 2.6. Statistical Analysis

The mean and standard deviation are presented for all the variables. The data were tested for normality using the Shapiro–Wilk test before parametric statistical analyses, and all data were found to be normally distributed. The interclass coefficients of correlation (ICC) for both the major oxygen kinetics parameters (i.e., time and rate constants) were calculated. For the correlational analysis, the rate constant (*k*) was the average of the two values across two visits. A Pearson correlation was analyzed between the average rate constant and peak oxygen uptake across visits. The Pearson correlation coefficient (r) values were interpreted as the following strengths of correlation: moderate, 0.30–0.49; large, 0.50–0.69; very large, 0.70–0.89; nearly perfect, 0.90–0.99; and perfect, 1.00 [33]. Differences in time and rate constants between the visits were tested using paired *t*-tests. Coefficients of variation (CV) for time constant and rate constant across the two visits were calculated and presented as group averages. Observed power (OP) was also reported when necessary. The level of significance was set at *p* < 0.05. SPSS Statistics for Windows software (v. 23.0; IBM SPSS, Armonk, NY, USA) was used for the calculations.

## 3. Results

All participants completed the all-out $\dot{V}$O_2_ peak test and fulfilled all three criteria for test validity. Peak physiological values attained during the test are presented in Table 1.

All participants tolerated the low-grade electrical stimulation well and had no problems or complications with the repeated blood flow occlusion protocol. The method provided a sufficiently reproducible m$\dot{V}$O_2_ time constant (ICC = 0.859; 95% confidence interval [CI], 0.632–0.950), although the reproducibility of the rate constant (ICC = 0.674; 95% CI, 0.265–0.877) was lower.

With the 15 s of electrostimulation, the m$\dot{V}$O_2_ was increased from baseline (pre-stimulation) values by no less than the recommended minimum of three times for each of the 22 measurements. After stimulation, the m$\dot{V}$O_2_ peak was 12.3 ± 2.1 times and 12.7 ± 4.4 times greater than the baseline value during the first and second lab visits, respectively (*p* > 0.05). The time constant of oxygen consumption recovery (29.6 ± 9.2 and 29.2 ± 8.9 s, *p* = 0.76) had a mean CV of 9%. The recovery rate constant (*k*) (2.33 ± 0.97 and 2.24 ± 0.79 min^−1^, *p* = 0.71) had a somewhat high (15%) mean CV.

**Figure 2:**
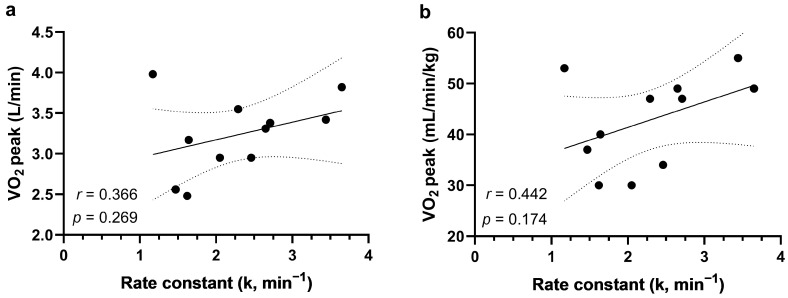
(**a**) Correlation between the absolute $\dot{V}$O_2_ peak and m. vastus lateralis oxygen uptake recovery kinetics rate constant (*k*); (**b**) Correlation between the $\dot{V}$O_2_ peak adjusted per kg of body mass and m. vastus lateralis oxygen uptake recovery kinetics rate constant (*k*). Dashed curves represent a 95 percent confidence interval. $\dot{V}$O_2_ peak, whole body peak oxygen uptake.

Correlation analysis revealed that the NIRS-derived marker of mitochondrial power (rate constant *k*) was not associated significantly with the cycling absolute $\dot{V}$O_2_ peak (*r* = 0.366, *p* = 0.27, OP = 0.50, Figure 2a) or $\dot{V}$O_2_ peak adjusted per kg of body mass (*r* = 0.442, *p* = 0.17, OP = 0.53; Figure 2b). Neither *k* was associated significantly with PPO (*r* = 0.376, *p* = 0.25, OP = 0.53).

## 4. Discussion

Since the reliability and validity of the relatively novel methodology of using NIRS with repeated limb occlusions in assessing muscle oxidative capacity remain under-investigated, the present study questioned: (1) whether indirectly measured skeletal muscle mitochondrial capacity is sufficiently reproducible in free-living active individuals arriving at the laboratory for repeated measurements over 2 days; and (2) if there is a correlation between skeletal muscle aerobic power measured indirectly using NIRS and the whole-body aerobic capacity. The method of indirect assessment of mitochondrial power emerged to be sufficiently reproducible for measurements of the m$\dot{V}$O_2_ recovery time and rate constants (despite the latter being somewhat less reproducible), while the correlations between the indirect markers of mitochondrial power and whole-body aerobic power were weak and not significant.

To the best of our knowledge, there are two reliable and feasible noninvasive techniques to assess skeletal muscle mitochondria power established thus far, namely to measure the speed of restoration of muscle phosphocreatine stores by means of magnetic resonance spectroscopy or the rate of recovery of muscle oxygen consumption via NIRS [32]. Both methods standardly require activation of the muscle with contractile activity, and resultant markers of mitochondrial power are closely associated [11], but NIRS offers an advantage over magnetic spectroscopy due to its higher portability and lower costs, making it more suitable for routine measurements. However, NIRS is limited to superficial muscles since current devices cannot measure the tissues deeper than ~3 cm beneath the probe, and this limitation is also relevant for subjects with substantial subcutaneous fat tissue reserves. In addition, NIRS could be troublesome in tissues beneath dark skin because of heavy tans, tattoos, etc.

The obtained ICC and CVs for day-to-day variability of vastus lateralis NIRS-derived mitochondrial power in our present study are of quite similar magnitude and strength to those reported by Hanna et al. [20], Pilotto et al. [28], Fennel et al. [23], Beever et al. [14], and Ryan et al. [10], investigating the same muscle on slightly different populations and using other protocols. Additionally, the CVs of the between-day time and/or rate constants of oxygen recovery kinetics have been reported to be quite similar for another leg muscle frequently investigated, the gastrocnemius [10,12,24,34]. In addition, the calculated *k* constants of our study are overall quite comparable with the above-mentioned studies. On the contrary, even in very active older individuals, the calculated vastus lateralis *k* was substantially lower [23], indicative of the declining aerobic capacity of the muscle with aging. Therefore, considering the minimal impact of these slight differences in study designs and/or protocols on the reproducibility of muscle oxidative capacity estimates, it supports the reliability of the method [14].

The NIRS-derived estimate of muscle mitochondrial power has been shown to discriminate successfully between athletic and patient populations [25], supporting the validity of the method. In combining two groups of low and high aerobic fitness young males, Lagerwaard et al. have shown a moderate but significant association of cycling $\dot{V}$O_2_ peak with NIRS-derived mitochondrial power of plantar flexors [13]. The same approach in females has revealed similar cycling $\dot{V}$O_2_ peak associations, with the aerobic power of plantar flexors and, somewhat weaker, with that of wrist flexors [17]. A moderate and significant correlation was found between cycling peak oxygen consumption and the NIRS-derived aerobic power of both the plantar flexors and knee extensors within a group of young participants with varied aerobic fitness levels [15]. Consequently, the validity and specificity of the method within a relatively homogeneous group of participants could be appropriately questioned.

Our present study in a more homogeneous subset of young males just fell short of finding a significant correlation between the NIRS-derived mitochondrial power of knee extensors and whole-body aerobic power measured as a cycling $\dot{V}$O_2_ peak. This is in accord with the findings of another study on young males, where NIRS-derived gastrocnemius muscle oxidative power was not correlated with $\dot{V}$O_2_ peak values measured on treadmills [12]. The participants had a somewhat higher (even if treadmill-determined) mean $\dot{V}$O_2_ peak of ~56 mL/kg/min (compared to our participants’ ~45 mL/kg/min), but their group was highly homogeneous (SD 3.4 mL/kg/min vs. our 9.2 mL/kg/min). Nevertheless, a study on young males and females with aerobic fitness levels similar to those in our present study, using voluntary contractions to activate the muscles rather than electrical stimulation, reported moderate yet significant associations between whole-body indices of aerobic power (including $\dot{V}$O_2_ peak and ‘anaerobic thresholds’) and NIRS-derived mitochondria power in the m. vastus lateralis, but not the gastrocnemius [14]. Finally, somewhat contrasting with the findings mentioned above, Azevedo et al. [35] reported a tight interdependence between NIRS-determined aerobic power in the m. vastus lateralis and pulmonary oxygen uptake across various intensities during recumbent cycling.

Even though one could be anticipating a significant correlation between the whole-body aerobic power ($\dot{V}$O_2_ peak) and that of the (presumably) most commonly used skeletal muscles by nonathletic recreationally active individuals during their daily activities and tasks such as ambulation, going up and down stairs, and sitting up and down, it did not materialize. Despite the fact that the muscle under investigation is heavily involved not only in daily activities but also during cycling [36,37], which was the exercise mode used for the $\dot{V}$O_2_ peak test protocol, the correlation between NIRS-derived muscle oxidative power and whole body (cycling) $\dot{V}$O_2_ peak did not emerge. An explanation for this result could be that because the muscular capacity to extract and utilize oxygen comprises only one of several links in the oxygen flow chain within the body, no stronger than a moderate association between it and the whole-body $\dot{V}$O_2_ peak could be observed or even expected. Indeed, there is a long-standing consensus that a multitude of factors affect the $\dot{V}$O_2_ peak, even if the capacity of the cardiovascular system to deliver oxygenized blood to the working muscle is, in most cases, considered to be the primary limitation [5]. Other factors, such as blood volume and composition (total hemoglobin mass and hemoglobin concentration), are no doubt also of critical importance and were likely to have quite different expression levels between individuals [5]. In support of the above interpretations, changes in the $\dot{V}$O_2_ peak with training in women have been shown not to be associated with the training-induced changes in the metabolic enzyme content of the trained muscle [38].

The obtained mitochondrial power has been derived from the dynamics of the (de)oxyhemoglobin content signal using an NIRS probe penetrating 1.5–2 cm deep into the soft tissue. The tissue layer in that depth for our relatively lean participants, even if not double checked with skinfold calipers or via ultrasonography, was more than likely superficial strata of m. quadriceps and, namely, its vastus lateralis head. Even if m. quadriceps and its vastus lateralis head in humans are not highly ‘aerobic’ in respect to their fiber type composition, especially on the superficial layers [39], being quite active during a lot of daily tasks and recreational sports (both apply to our young recreationally active participants) most likely offsets this ‘drawback’ by adding more mitochondria and oxidative enzyme power. Therefore, the derived mitochondria power could be expected to be somewhere around the middle region of the spectrum of the skeletal muscles, as well as those involved in cycling.

Stationary cycling obviously involves many more muscles than just the vastus lateralis head of m. quadriceps and also more muscle groups than just m. quadriceps [36,37]. The correlation between the vastus lateralis and $\dot{V}$O_2_ peak would most likely be stronger if, instead of cycling, one would use unilateral knee extensions performed in a well-controlled fashion using isometric contractions within the dynamometer [23]. Such reasoning is based on the notion that the specific primary factors limiting the $\dot{V}$O_2_ peak are dependent on the muscle mass involved in an exercise task and, therefore, the exercise type and mode themselves. With exercises involving a limited number of muscle groups, small muscles, and overall small muscle mass, the primary factors in achieving a high $\dot{V}$O_2_ peak would be largely ‘peripheral’, that is, the quality of the muscle tissue involved per se (content and activity of the mitochondria enzyme activity in the muscle fibers; muscle fiber mitochondria characteristics such as size, distribution, and total volume; muscle capillary network; and other determinants such as connective tissue content). However, such isolated exercises, even if testing were performed using well developed protocols and performed simultaneously on both legs, would evoke much lower $\dot{V}$O_2_ peak values, which would be less ecologically relevant for most subject populations.

While not in our current study, NIRS-derived muscle mitochondria power has been shown to be associated with ergometric test performance, e.g., [14,23]. One study has even found this indirect marker of mitochondrial power, among multitudes of traditional physiological indices, to be the best predictor of prolonged time trial performance in the moderate altitude lab [22], leaving us to speculate for now whether this would translate to other settings and populations.

Insufficient muscle contraction-induced mitochondrial activation (i.e., low m$\dot{V}$O_2_) could lead to a low rate constant that misrepresents the “true” oxidative capacity of the muscle, which could be a significant factor resulting in the poor test–retest reproducibility of the rate constant [24]. Increased muscle metabolic activity can be induced by voluntary activation of the muscles, which has been applied successfully [26], or by electrostimulation. With the electrostimulation protocol used in the present study, the oxygen consumption rate within the musculus vastus lateralis tissue increased on average ~12.5 times, as estimated from the NIRS signal dynamics during the recovery period with repetitive occlusions on a relaxed muscle. The oxygen consumption rate in each participant for a total of 22 measurements increased by no less than three times, a recommended minimum for the successful application of NIRS in estimating muscle mitochondrial capacity [10,20,26]. Therefore, the activation of the skeletal muscle with mild or short stimulation was sufficient to fulfill the requirements of the protocol.

One of the study limitations was that no reproducibility analysis was conducted on the whole-body aerobic capacity marker ($\dot{V}$O_2_ peak). It must be acknowledged that several $\dot{V}$O_2_ peak tests within a short time could have yielded a more accurate estimate of the whole-body aerobic capacity of the participants and a value closer to the “true” $\dot{V}$O_2_ max. However, scheduling repetitive lab visits for such tests is impractical due to the inconveniences it puts on the participants, and doing so would barely improve the precision of the estimate of the relationships. A second limitation is related to the small sample size in this study. Specifically, this small sample size could have prevented us from obtaining high statistical power for our correlation analyses. Therefore, these correlations should be interpreted with caution. However, these moderate-to-large correlations are in line with previous research [12,14,23]. In addition, because only young, healthy, lean males were recruited as participants, the ability of the NIRS method to determine muscle aerobic power must be proven for validity in other populations, including those with high skin melanin content, those possessing thick layers of subcutaneous fat, or those with limited peripheral blood flow, as found in those with diabetic arterial lesions. These and other factors affecting the applicability of NIRS for muscle mitochondrial oxidative capacity measurements have been extensively outlined by Adami and Rossiter [25].

Research perspectives. The method remains to be validated in specific populations, such as high-performance endurance athletes. Also, it would be highly desirable to have more automated processing of the signals, which would also help to derive the oxygen recovery constant and overall results of estimated mitochondrial power from single or multiple muscles faster.

## 5. Conclusions

In conclusion, the indirect NIRS-based estimation of skeletal muscle mitochondrial power demonstrates good overall reliability. However, the derived marker of muscular aerobic capacity did not show a significant correlation with the whole-body aerobic capacity measured during exercise involving the investigated large leg muscles. Therefore, NIRS-derived estimations of skeletal muscle mitochondrial power and “whole body” aerobic capacity seem to represent two different entities and should not be regarded interchangeably when analyzing aerobic fitness.

## Figures and Tables

**Table 1:** Peak physiological values attained during cycling aerobic capacity test.

**PPO, W**	305.4 (32.7)
$\dot{V}$ **O_2_ peak, mL/kg/min**	44.7 (9.2)
**HR peak, bpm**	180 (12)
**VE, L/min**	125 (21)
**RER**	1.19 (0.04)

PPO, peak power output; $\dot{V}$O_2_ peak, peak oxygen uptake; HR peak, peak heart rate; VE, peak pulmonary ventilation; RER, peak respiratory exchange ratio. Data are mean (SD) (*n* = 11).

## Data Availability

Data are contained within the article.
